# A pilot study of metabolic fitness effects of weight-supported walking in women with obesity

**DOI:** 10.1371/journal.pone.0211529

**Published:** 2019-02-20

**Authors:** Ellen M. Godwin, Anthony D. Uglialoro, Andaleeb Ali, Leah Yearwood, Mary Ann Banerji, John G. Kral

**Affiliations:** 1 Department of Physical Therapy, Long Island University, Brooklyn, New York, United States of America; 2 Department of Orthopedics/Rehabilitation, SUNY Downstate Medical Center, Brooklyn, New York, United States of America; 3 Department of Surgery, SUNY Downstate Medical Center, Brooklyn, New York, United States of America; 4 Department of Medicine, SUNY Downstate Medical Center, Brooklyn, New York, United States of America; University of Missouri Columbia, UNITED STATES

## Abstract

**Background:**

This is an exploratory pilot study of novel technology enabling people with mobility disability to walk with minimal effort, in the “sedentary range”. The study’s premise is that impairment of the leading physical activity of daily living, walking, is a major contributor to a dysmetabolic state driving many prevalent “civilization diseases” associated with insulin resistance.

**Methods:**

We explore within-subject changes in standard oral glucose tolerance (OGT) tests including metabotropic molecules after 22 twice-weekly, 30-minute bouts of weight-supported light-moderate physical activity in 16 non-diabetic obese, otherwise healthy, reproductive-age, volunteer women walking on an “anti-gravity” lower-body positive pressure (LBPP) treadmill.

**Results:**

Subjects had reference base-line fasting plasma glucose and triglycerides (TG) but 2-hr OGT insulin levels of 467 ± 276 pmol • liter^-1^ (mean± S.D.) indicating nascent insulin resistance, compared to post-study 308 ± 179 (p = 0.002). Fasting TG decreased from 0.80 ± 0.30 mmol • liter^-1^ to 0.71 ± 0.25 (p = 0.03). Concomitantly plasma total ghrelin decreased from 69.6 ± 41.6 pmol • liter^-1^ to 56.0 ± 41.3 (p = 0.008). There were no statistically significant changes in body weight or any correlations between weight change and cardiometabolic markers. However, there were robust positive correlations between changes among different classes of peptides including C-reactive protein–Interleukin 6, leptin–adiponectin, β-endorphin–oxytocin and orexin A (r ^2^ = 0.48–0.88).

**Conclusion:**

We conclude that brief, low-dose physical activity, walking on an anti-gravity LBPP treadmill may improve cardiometabolic risk, exhibiting favorable changes in neuro-regulatory peptides without weight loss in people with problems walking.

## Introduction

Mobility disability, the leading form of disability, is increasing in step with epidemic obesity [1, 2]. Although life-style interventions require sustained moderate—high levels of physical activity [3, 4], persons with overweight/obesity are physically impaired in their ability to walk, the leading energy-consuming activity of daily living (ADL). Thus, life-style change regimens with diet and exercise recommendations, the primary therapeutic and preventive interventions for numerous degenerative diseases including obesity, osteoarthritis and cardio-pulmonary insufficiency, are rarely maintained long enough to be effective over the long term [3, 5, 6] owing to the fact that exercise is hard and painful.

Availability of a NASA developed anti-gravity treadmill offloading body weight using lower body positive pressure (LBPP) [7], prompted us to test whether use of this treadmill would engender known positive metabolic effects of increasing physical activity [8]. We recruited a convenience sample of 16 sedentary, obese, non-diabetic, reproductive-age volunteer Black women, culturally disinclined to lose weight [9]. Therefore we chose an activity level well below life-style guidelines [10], enabling comfortable, safe ambulation according with ADL levels of painless exertion within the “sedentary range” unlikely to cause weight loss [4; 11; 12; 13].

Most interventions are implemented late in cumulative disease processes when the magnitude and the rapidity of recommended interventions exceed the speed with which calories have been stored [14; 15]. Such relatively large and rapid changes (low calorie diets, moderate-high intensity exercise) cause counter-regulatory ‘stress’ as maladaptive non-homeostatic dysautonomic responses, similar to mechanisms of insulin resistance [16]. Whereas exercise (exertion) is beneficial in physically fit individuals [17], dose-response levels are circumstantial and highly variable owing to intrinsic and environmental influences.

Although the “sedentary range” demonstrates that increasing physical activity is uncoupled from appetite, interpreted as “dysregulation of energy intake” supporting recommendations to exceed the sedentary range [13], we posit that uncoupling of appetite and food intake from exertion might be beneficial. Lower levels of physical activity within the sedentary range, by being undetected, imperceptible or “dysregulated”, might expend sufficient energy without exertion that challenges lean body mass [14] or sensitizes appetite [12;18;19]. At the same time, accumulating evidence supports positive effects of low intensity activity [20–23]. Whereas most guidelines propose moderate to high intensity exercise to lose weight, there is little research investigating metabolic effects of lower levels of energy expenditure without weight loss [13; 20, 21] and, to our knowledge, none on brief, low-amount, activity-of-daily living level ambulation.

This pilot study implies that an investment of 30 minutes of lower-body pressure (weight-offloading) walking twice weekly for 10–12 weeks is sufficient to significantly reduce nascent insulin resistance independent of weight change in non-diabetic reproductive-age Black women.

## Methods

### Subjects

Volunteers among hospital employees and their families (not related to or supervised by the investigators) were recruited through word-of-mouth at SUNY Downstate Medical Center, a convenience sample. They were offered participation in a study of “metabolic fitness” defined as “the ability of the body to use energy from dietary sugars and fats”. Eligible were otherwise healthy overweight–obese (BMI 28–50; mean ± SD: 35 ± 7), weight-stable, untrained, non-diabetic, pre-menopausal Black women aged 18 to 56 years (40 ± 11) (S1 Table).

Participants were willing and able to commit to 2–3 times weekly 30-minute bouts of exercise on the weight-supporting treadmill for 12 weeks (more than 20 bouts) comfortably and painlessly using the treadmill. They were not compensated but received a thorough metabolic work-up described below, signing consent forms covering all aspects of this study approved by the Institutional Review Board of SUNY Downstate Medical Center specifically for this study (IRB# 12–044). Investigators blinded to accrued phenotypic data performed all assays and measurements, treadmill supervision and statistics.

Exclusion criteria were shift-work and strenuous work-related functions, participation in dieting or exercise programs the previous 3 months, surgical treatment of obesity, being pregnant or planning pregnancy, being smokers, taking medications known to affect energy balance or having musculo-skeletal or other conditions incompatible with using the treadmill. We excluded subjects with known diabetes, hypertension and dyslipidemia and those using psychotropic medications potentially affecting appetite regulation, oral contraceptives or steroids of any type. They consumed no or only minimal alcohol.

#### Baseline history, physical and activity assessment

A structured 45-50-minute interview based on questionnaires exploring demographic and socio-economic factors, food insecurity, household stressors and a thorough medical history was conducted. Anthropometric measures included weight, height, BMI, waist and hip circumferences. Blood pressure and heart rate were measured after subjects had been seated for 5 minutes. Taking into consideration the cultural habits and beliefs of our population, there was no “weighing in” or mention of body weight, diet or dieting during visits to the laboratory. Most subjects were administrators engaged in typically sedentary work. Daily activity was exclusively measured pre- and post-study using a wrist-worn Fit-Bit accelerometer for two 3-day periods one of which included one non-working weekend day (S1 Methods).

### Fasting morning blood and oral glucose tolerance tests

An in-dwelling ante-cubital venous catheter was placed with the patient seated in a comfortable recliner. At time 0 of a standard 75 g oral glucose tolerance test (OGTT) blood was drawn for determination of glucose and insulin, HOMA-IR, GLP-1, GIP, free fatty acids (FFA), C-peptide, fasting C-reactive peptide (CRP), Interleukin-6 (IL-6), TNFα, leptin, total ghrelin, total adiponectin, GIP, glucagon, triglycerides (TG), HDL-cholesterol, oxytocin (OXT), β-endorphin and orexin A (ORA). During OGTT at 15, 30, 60, 90 and 120 minutes blood was drawn for glucose, insulin, C-peptide, GLP-1 and FFA, allowing calculations at 2 hours and area under the curve (AUC). Blood chemistry details are provided in Supporting Methods (S2 Methods).

### Weight-supporting treadmill

The lower-body positive pressure (LBPP) treadmill (AlterG) [24] consists of a stationary heavy metal frame with a movable gantry that can be raised to the level of a user’s waist and is attached to an inflatable clear-plastic malleable “skirt” surrounding the electric treadmill. (S1 Fig) The waist of the skirt is encircled with one portion of a zipper corresponding to a zipper around the waist of neoprene shorts, worn by the subject. The treadmill’s mechanism covertly measures the person’s weight, enabling determination of a percentage of body weight to be off-loaded by treadmill pump inflation increasing pressure on the lower body. The maximum pressure exerted is equal to that of standing in water in a pool (~ 1 psi; 51.7 mmHg; 70 cm H_2_O; 6.9 kPa) [25].

### Treadmill protocol

Off-loading was set at 40% reduction of body weight, subjects thus walking on the treadmill at 60% of their weight throughout the study, a level approximating long-term weight loss after successful metabolic surgery. Speed set by the subject, by adjusting treadmill speed on the dashboard, allowed completion of a 30-minute session. Initial incline was set at 0°. Subjects were encouraged to increase the speed with time and staff gradually increased the incline every 3 sessions to 3, 5, 7, and a maximum of 10%. Time, distance, speed, treadmill incline and % weight offloaded at the 25-minute mark was recorded after each session. After completion of 20–24 sessions OGTT with fasting blood tests, similar to before the exercise regimen, were performed at least more than 24 hours after the last session, sufficient to rule out acute effects of this brief (30-minute) low-intensity, infrequent (twice weekly) incremental exertion.

#### Energy expenditure

We calculated metabolic equivalents (METs) of energy expenditure according to standard equations [26], but adjusted for weight in our study of weight-supported ambulation. We used the Harris-Benedict Equation for RMR to improve the accuracy of MET estimates to determine “corrected METs” when offloading body weight with the LBPP treadmill [27].

Corrected METs, kilocalories (kcal) and Watts (W) expended per session according to ACSM formulas, total energy expended per week as MET minutes and kcal expenditure were used to compare our effort intensity at 60% body weight with CDC and ACSM exercise recommendations at full body weight [28] adding up total energy expended by each subject during all sessions (S1 Methods).

### Statistics

Data are expressed as means ± standard deviations (SD), except as noted. Within-subject differences pre- and post-intervention were compared using paired sample t-tests or Wilcoxon’s rank-sum tests based on a one-sample Kolmogorov-Smirnov test against a normal distribution. The exploratory nature of this pilot study with 16 subjects precluded co-varying for multiple variables in the pre-post intervention analyses. Similarly, we have not corrected for multiple testing. We calculated Pearson correlation coefficients between relevant variables and used Spearman correlations when relationships were not linear owing to the variance in this relatively small population. Statistical analyses used SPSS version 23 (SPSS, Chicago, IL).

## Results

### Baseline characteristics and proteomics

According to our inclusion and exclusion criteria and the characterization in Methods, these 16 subjects were overweight/obese and sedentary but were not diabetic, dyslipidemic or hypertensive. Mean weight was 93.9 ± 13.6 kg, waist circumference 108 ± 22 cm and blood pressure 113/74 mmHg (S1 Table). There were no statistically significant correlations between BMI and baseline molecules. In addition to traditional diabetes and dyslipidemia biomarkers, we analyzed an array of incretins, inflammatory cytokines, adipokines and neurometabolic peptides individually known to be responsive to exercise and lower-body positive-pressure. We found several novel baseline correlations between these different classes of peptides, relevant to our post-study outcomes (Table 1A and 1B).

**Table 1:** Baseline correlations of plasma A. cardio- and B. neuro-metabolic and appetitive molecules.

*A*			
Glucose AUC*		r	p
	Glucose, 2-hr +	0.648	0.007
	Insulin AUC +	0.671	0.004
	C-peptide, AUC +	0.800	0.0001
	GIP	0.506	0.046
	Ghrelin	0.675	0.004
	Orexin-A	-0.558	0.025
	Oxytocin	-0.527	0.036
	Beta endorphin	-0.504	0.047
*Insulin*			
	GLP-1	0.704	0.002
	Glucagon	0.838	0.0001
	GIP	0.581	0.018
	Triglyceride	0.778	0.0001
*GLP-1*			
	Glucagon	0.802	0.0001
*Interleukin-6*			
	Insulin	0.574	0.020
	C-Peptide	0.474	0.06
	TNF-Alpha +	0.591	0.016
*Triglyceride*			
	TNF-Alpha	0.508	0.045
	Interleukin-6	0.482	0.058
	Glucagon	0.648	0.007
*HDL-cholesterol*			
	Ghrelin	0.621	0.01
	Glucagon +	0.572	0.021
*B*			
*Leptin*			
	Orexin A	-0.512	0.042
	Oxytocin	-0.578	0.019
	Beta endorphin	-0.624	0.010
*Ghrelin*			
	Adiponectin +	-0.453	0.078
*Orexin A*			
	Oxytocin	0.889	0.0001
	Beta endorphin	0.705	0.002
*Beta endorphin*			
	Oxytocin	0.821	0.0001

*2-hr after oral glucose (OGTT); AUC: area under curve

** Correlations are Pearson unless noted; + Spearman Correlation

### Physical activity

Subjects ambulated for 30 minutes 2–3 times weekly (mean 2.2) for 11 weeks on the Alter-G treadmill at 60% body weight without complications. They walked/jogged a mean distance of 2 miles (3.2 km) each session at a mean rate of 4.3 mph. The weekly addition of 4 miles corresponded to a 19% increase in weekly ambulation from 21 to 25 miles. The average level of intensity was 8.5 METs calculated as corrected METs at 60% body weight, representing the lower end of recommended weekly expenditure (Table 2) [28].

**Table 2:** Energy expenditure during twice-weekly LBPP treadmill sessions (mean ± SD).

Speed per session (mph)	4.3 ± 1.2
Distance per session (miles)	2.0 ± 0.55
Treadmill Grade (%)	6.4 ± 0.4
Days for completion of study	80 ± 28
Number of sessions	22 ± 2
Intensity (Corrected METs) ^1^	8.5 ± 2.8
Corrected MET^1^ minutes per week	512 ± 131
Total energy expended (MET minutes)^1^	5874 ± 1725

^1^at 60% of body weight (BW) [Ref. 27]

### Cardio- and neuro- metabolic changes

There were no statistically significant before-after mean differences in body weight, waist circumference or blood pressure. Eight subjects gained (range: 1.0–7.0 kg) and 6 lost weight (range: 2.2–9.5 kg), two exhibiting no change. Subjects, who had lost weight, disclosed *post hoc* during informal follow-up interviews that they had, on their own, dieted during and for several months following the study, although instructed to “eat as usual”. Nevertheless, there were no statistically significant differences in outcomes between those with weight loss and those with weight gain. The mean duration between last exercise bout and blood testing was 9.8 days (range 28 hours—44 days), with no statistically significant relationships between time post last session and any outcome described in the following sections.

#### Dynamic Metabolic Testing (OGTT)

**Fig 1:**
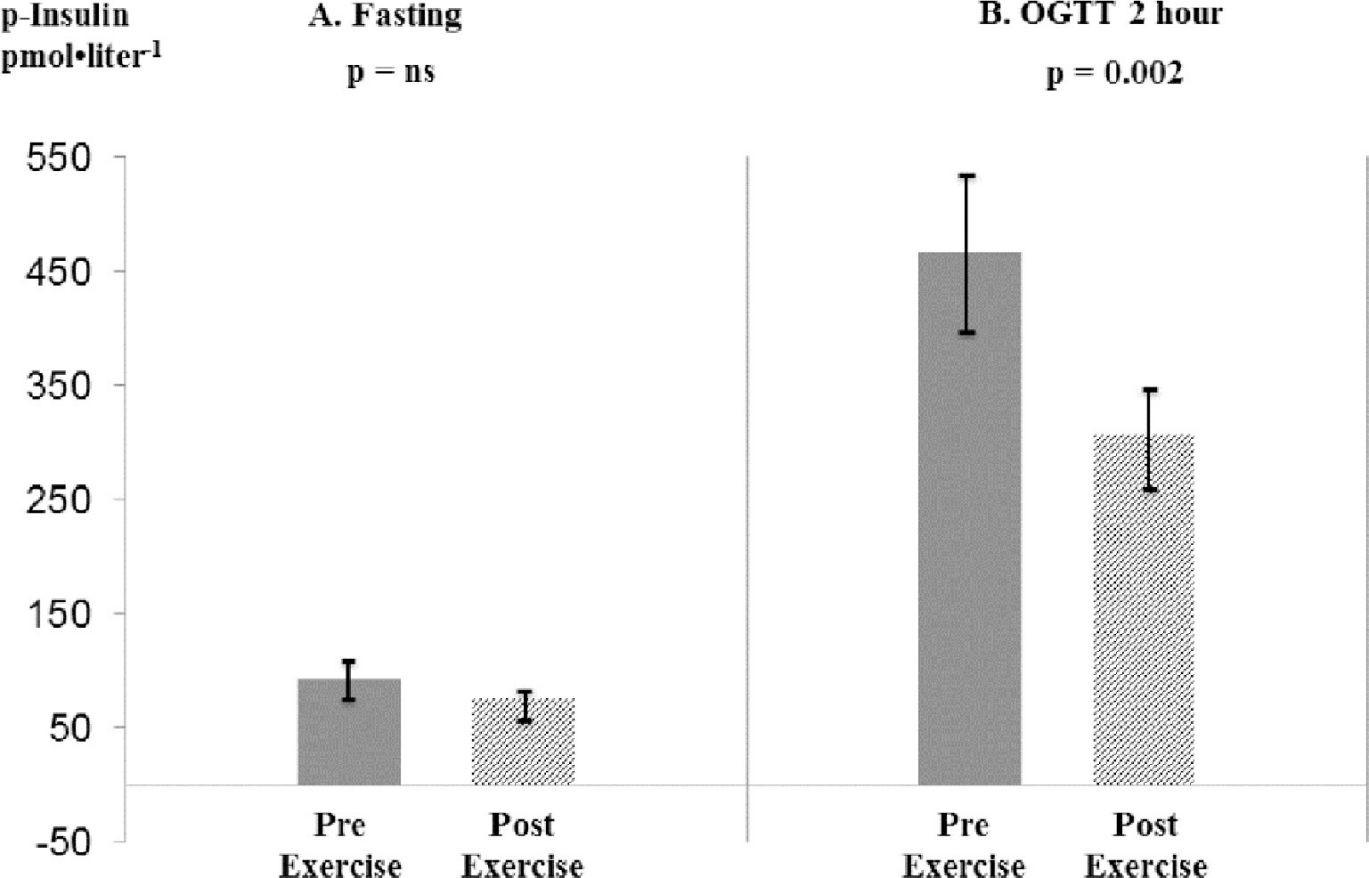
**A. Fasting p-insulin. B. OGTT 2-hour p-insulin pre- and post- weight supported ambulation** (n = 16; mean ± s.e.m.).

After completion of the program, the 75-gram oral glucose challenge showed statistically significant decreases in both 2-hour (Fig 1B) and incremental *insulin* responses (34% [p = 0.002] and 15% [p = 0.022] respectively) concomitant with a significant 15% decrease in 2-hour C-peptide (p = 0.033) and trend towards a 19% decrease in 2-hour GLP-1 (p = 0.06;Table 3), together supporting reduced insulin resistance. There were only marginal, not statistically significant decreases in fasting, 2-hour and incremental *glucose* area.

**Table 3:** Changes in plasma cardio-, neuro-metabolic and appetitive molecules before (Pre-) and after (Post-) bodyweight-supported walking (mean ± SD).

		Pre	Post	Change	P
**2 Hour Post Oral Glucose (OGTT)**					
Glucose	mmol.liter^-1^	6.4±2.2	6.0±1.4	-0.4±1.1	0.19
Insulin	pmol.liter^-1^	467 ± 276	308 ± 179	-160 ± 174	0.002
C-Peptide	nmol.liter^-1^	1.21 ± 0.46	1.03 ± 0.41	-0.18 ± 0.31	0.03
GLP-1	pmol.liter^-1^	12.57 ± 5.3	10.18 ± 5.1	-2.39 ± 4.7	0.06
**Fasting**					
Glucose	mmol.liter^-1^	5.0 ± 0.9	4.9 ± 0.9	-0.1 ± 0.5	0.17
Insulin	pmol.liter^-1^	92 ± 59	76 ± 46	-17 ± 70	0.36
Triglyceride	mmol.liter^-1^	0.80 ± 0.30	0.71 ± 0.25	-0.10 ± 0.16	0.03
Interleukin-6	pg.ml^-1^	7.37 ± 4.87	11.62 ± 16.27	4.30 ± 11.8	0.17
Adiponectin (total)	µg.ml^-1^	11.7 ± 4.6	12.0 ± 5.1	0.3 ± 2.3	0.61
Leptin	µg.liter^-1^	31.0 ± 14.1	34. 6 ± 12.8	3.57 ± 7.7	0.08
Ghrelin	pmol.liter^-1^	69.65 ± 41.60	55.97 ± 41.27	-13.68 ± 18.0	0.008
Orexin-A	pmol.liter^-1^	150 ± 42	187 ± 91	37 ± 110	0.20
Oxytocin	pmol.liter^-1^	89.3 ± 37.2	113.3 ± 85.8	24.0 ± 99.1	0.35
Beta endorphin	pmol.liter^-1^	103 ± 40	128 ± 58	25 ± 67	0.16

#### Fasting cardiometabolic and inflammatory molecules

Several differences in fasting plasma parameters were noted comparing before to after program completion. Glucose, in the normal range decreased slightly (p = 0.17) while there was a 19% numerical, not statistically significant reduction in fasting plasma insulin (p = 0.36) (Fig 1A). There were no statistically significant changes in adiponectin which increased 2.6% (p = 0.61) or decreases in C-peptide, GIP and glucagon (S2 Table), with no significant change in fasting GLP-1. There was a trend toward increased leptin (12%; p = 0.084).

Triglycerides, not elevated in any subject at baseline, decreased 11% (p = 0.03) (S2A Fig) with discordant changes in free fatty acids. HDL cholesterol increased minimally (S2 Table).

Changes in inflammatory cytokines varied between subjects according to base-line status, undetectable in some, divergent in others. C-reactive protein (CRP) was elevated in 7 subjects in whom it decreased by 25% (p = 0.052). TNFα levels decreased in 7 subjects with detectable levels pre-exercise. Anti-inflammatory Interleukin-6, detectable at baseline in 8 subjects (mean 7.4 ± 4.9 pg/ml) increased 58%, not statistically significant post study (mean 11.6 ± 16.3; p = 0.170).

#### Fasting neuropeptides

Plasma ghrelin had decreased by 20% at the end of the study (p<0.008; S2B Fig) whereas there were no statistically significant changes in orexin A, which increased numerically 25% (p = 0.169), oxytocin 27% (p = 0.348) and β-endorphin 24% (p = 0.156). These relatively large within-subject numerical increases were statistically insignificant by 2-tailed t-tests owing to the relatively small numbers of subjects.

#### Correlations between before-after changes

There were no statistically significant correlations between body weight change or total energy expended (MET minutes) and decreases in OGTT glucose, 2-hr insulin, C-peptide, fasting triglycerides or ghrelin. Significant correlations were present between total MET minutes and decrease in glucoregulatory 2-hr GLP-1 (r = - 0.679; p = 0.004) and the numerically small (0.3 μg/ml) increase in adiponectin (r = 0.471; p = 0.066). Changes in 2-hour GLP-1 were unrelated to changes in other appetitive peptides (ghrelin, Orexin A, β-endorphin).

**Fig 2:**
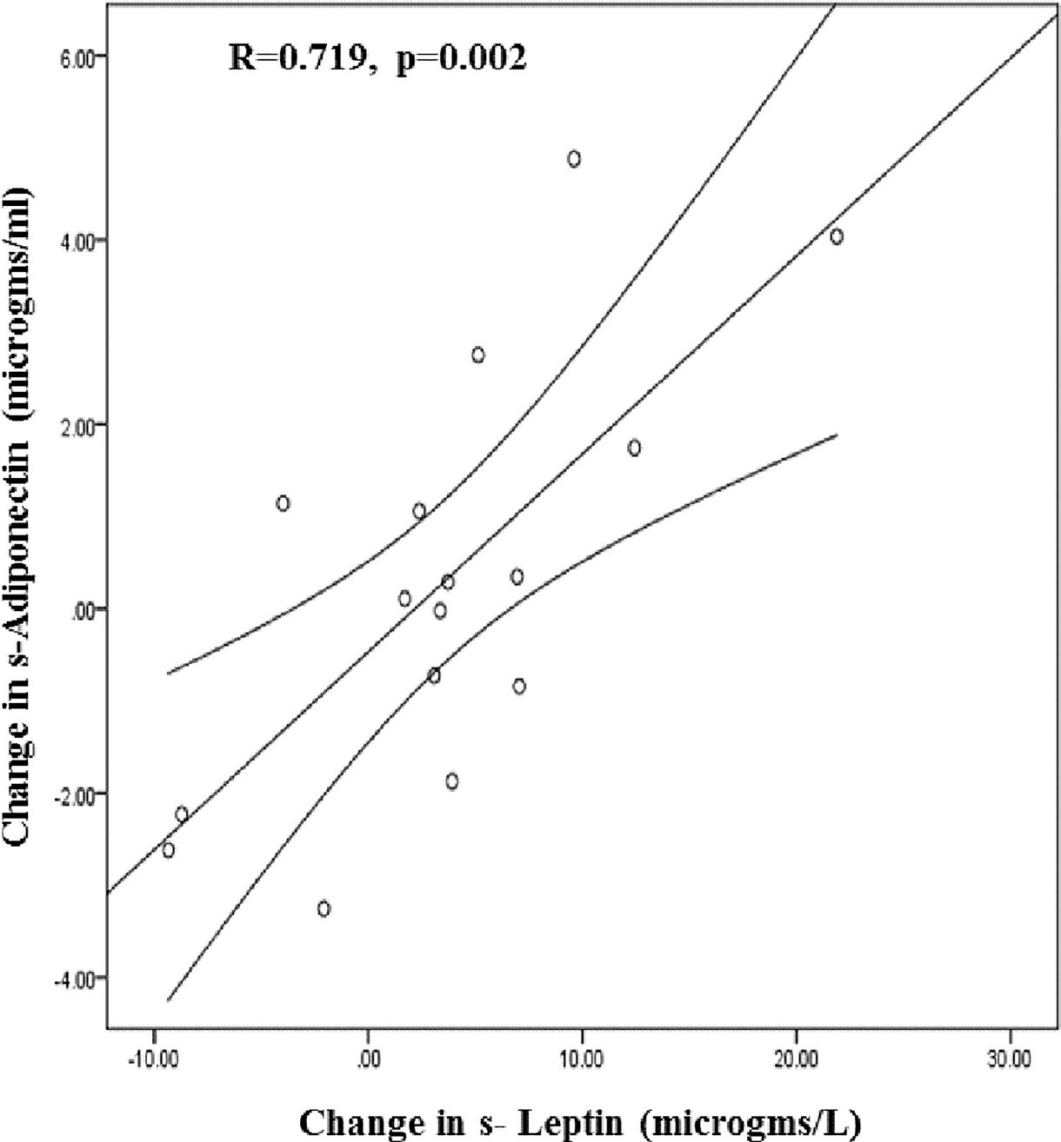
Pearson correlation of changes (Δ) in s-adiponectin and s-leptin, pre- and post- weight supported ambulation. [Y = 0.47+0.21X; r = 0.719; p = 0.002. Fit line 95% CI for the mean. (n = 16)].

Although parametric statistical analyses did not detect significant before-after changes in many peptides, the changes were remarkably highly and consistently correlated. Intercorrelations between drops in gluco-regulatory 2-hr glucose, related to TNFα, and 2-hr insulin and C-peptide were significant; 2-hr insulin, in turn was highly correlated with rises in β-endorphin, orexin A and oxytocin (Table 4). Baseline levels of leptin, marginally correlated with 2-hr insulin (r = 0.38; p = 0.143), predicted the reduction in 2-hr insulin (r = 0.566; p = 0.022). Significant correlation between increases in fasting leptin and adiponectin (r = 0.718; p = 0.002; Fig 2) reflected improved insulin sensitivity concordant with significant reductions in insulin and C-peptide. HDL-cholesterol change correlated with FFA AUC, leptin and adiponectin but not with any inflammatory cytokines (Table 4).

**Table 4:** Correlations between changes in cardio- and neuro-metabolic molecules.

	r	p*
Glucose, 2-hr*		
Insulin, 2-hr*	0.445	0.084
TNF α	0.631	0.009
Leptin	0.482	0.059
Ghrelin	0.457	0.076
Orexin A	-0.502	0.048
Oxytocin	-0.422	0.103
Beta Endorphin^**+**^	-0.517	0.037
Insulin, 2-hr*		
C-Peptide, 2-hr^**+**^	0.538	0.031
Triglyceride	0.357	0.174
Leptin	0.475	0.063
Orexin A	-0.594	0.015
Oxytocin	-0.690	0.003
Beta Endorphin	-0.487	0.056
C-Peptide, 2-hr*		
C-Reactive Protein	0.451	0.079
GLP-1, 2-hr*		
Glucagon	0.555	0.026
IL-6	0.462	0.071
**GIP**		
Glucagon	0.580	0.019
C-Reactive Protein	0.532	0.034
**HDL**		
Leptin^+^	0.547	0.033
Adiponectin^+^	0.587	0.015
**CRP**		
IL-6^+^	0.689	0.003
**Adiponectin**		
Leptin	0.719	0.002
**Orexin A**		
Oxytocin	0.935	0.0001
Beta Endorphin	0.737	0.001
**Oxytocin**		
Beta Endorphin	0.783	0.0001

* 2 hours after oral glucose tolerance test (OGTT)

**AUC area under curve of OGTT**

^**+**^ **Spearman correlation; Pearson correlations unless noted**

We found previously not reported highly significant correlations between changes in before-after exercise *neuro-endocrine* peptides in agreement with the pre-exercise correlations and unrelated to total energy expended, weight changes or any phenotypic data (Table 4).

Altogether, these findings suggest before-after reductions in gluco-regulatory molecules reflected in correlations between glucose, insulin, C-peptide and incretins during OGTT. These reductions correlate with inflammatory markers, adipokines and neuroregulatory, appetitive peptides.

## Discussion

### Physical activity

#### Cardio-metabolic factors

The preliminary findings of this unique study challenge the premise of the preponderance of studies and guidelines for treating and preventing diabesity, namely that body weight is of primary importance for the disease and requires intermediate to high levels of frequent physical activity to improve performance and to cause weight loss [29]. Our sedentary subjects did not lose weight from the low level of exertion in the sedentary range and, indeed, there were no associations between energy expended or weight loss or gain and improved cardio-metabolic metrics such as insulinemia, glucose tolerance markers and plasma triglycerides earlier described in literature supporting diet and exercise guidelines.

Robust reductions in plasma triglycerides in the normal range in our Black subjects, normally with lower levels than those with other geographic ancestry [30,31], are significant in the context of increased recognition of low-moderate triglyceride levels as cardio-metabolic risk factors [32] furthermore supporting the need to redefine blood lipid standards for Black people [31]. Hyperinsulinemia has long been known as a driver of hepatic triglyceride synthesis [33], which accords with our present finding of concomitant reductions of OGTT insulin and fasting plasma triglycerides after our low intensity ambulation which otherwise is found after intensive exercise [34, 35]. Our related findings of correlations between fasting leptin and adiponectin and reductions of 2-hr insulin and C-peptide reflecting improved insulin sensitivity might imply less resistance of leptin receptor exposed to increased levels of leptin.

Our findings regarding *inflammatory markers* are similar to those of other studies of obese populations, although we generally excluded subjects with advanced disease sufficient to manifest detectable elevations of cytokines measured by routine methods. Our subjects were hyperinsulinemic, without impaired fasting glucose (with the exception of 3) or glucose tolerance, a pre-cursor to pre-diabetes. Nevertheless 7 subjects had detectable elevated CRP and 7 had elevated TNFα, all of whom reduced their levels post study, implying reduced inflammation associated with adiposity Three subjects did have impaired glucose tolerance (IGT) which normalized post-study independent of weight changes. These three were among the subjects that increased post-study levels of the anti-inflammatory IL-6, known to be beneficially elevated by exercise [20, 36].

#### Neuro-endocrine peptides

Remarkably, the low amount of low intensity activity combined with mild lower-body positive pressure was associated with effects on a wide array of cholinergic regulatory central and peripheral peptides. There was a substantial statistically significant reduction in fasting levels of orexigenic ghrelin, a rapid sensor of fluctuations in nutrient stores that was not associated with statistically insignificant increases in ‘satiety’ peptides leptin and β-endorphin or orexin A shown in exercise studies [37–41]. Owing to the large statistically insignificant related increases in plasma orexin A, oxytocin and β-endorphin in our underpowered study, we performed a median split analysis, which exhibited statistically robust differences (all p<0.01; not shown), consistent internally and with exercise and baro-physiology literature. Since glucose is the primary substrate in brain, skeletal muscle and whole-body metabolism our finding of a strong relationship between the reduction of glucose area under the curve and ghrelin reduction (p<0.004) is interpreted as a primary response to ambulation preceding transition to lipid and amino acid utilization in the sedentary range. In addition, there were no relations between fasting plasma ghrelin and fasting and glucose-stimulated levels of the pleiotropic neuro-endocrine brain-gut peptide GLP-1. Although it is synthesized in the ileum and mediates incretin effects associated with insulinotropic pancreatic β-cell secretion exhibited in this study, GLP-1 is also known for its central effects suppressing appetite and increasing neurogenesis [20, 42], here demonstrating discordance between its peripheral and central effects, possibly related to improved GLP-1 receptor function. Fasting and OGTT 2-hr GLP-1 have been shown by others to respond differentially to exercise [41].

The robust correlations between leptin and β-endorphin, orexin A and oxytocin and among these latter peptides are intriguing, given their respective roles in energy balance, neurogenesis and mood [43–46]. Although not the subject of this study, documented beneficial effects of exercise on anxiety and mood disorders [21, 47, 48] might be expected, related to our neuro-peptide changes after low-intensity exercise, especially in the absence of pain, a powerful mediator of mood. Informally all of our subjects have later spontaneously expressed their enjoyment of this exercise, especially those with experience with conventional treadmills. Given the problem of adherence to conventional exercise programs, it is conceivable that LBPP activity might be useful in the context of neuro-psychiatric studies in subjects with mobility disability [18]; the brain-gut peptides in this study participate in cholinergic circuits that modulate appetite suppression on downstream targets in the hypothalamus [49] and affect pancreatic structure, function and release of neurotrophic peptides other than GLP-1.

Our heterodox findings of improved glucoregulation with triglyceride decrease, mild effects on cytokines and adipokines but a robust effect on the orexigenic peptide ghrelin in the absence of weight loss accompanying brief, ambulation raises questions about putative mechanisms. We speculate that improvements in substrate utilization such as glucose disposal, lipolysis or “protein sparing” are not likely to be primary determinants, but rather synergistic with autonomic nervous system activation/conditioning (“stress buffering”) [17] and/or release of key molecules through mild compression and use of lower extremity muscles known to have beneficial cardiovascular [50, 51], angiogenic and metabolic effects associated with restored autonomic balance.

### Baro-physiology

Standing, *per se* improves cardiometabolic risk when used as a break in sedentary activity [21, 52] as do exercise “snacks” [53]. The compression exerted by the lower-body positive pressure treadmill during 40% off-loading is similar to standing in a swimming pool unrelated to muscle use. We have demonstrated decreased heart rate during weight offloading during standing [54], recently shown by others during increasing levels of exertion during LBPP [55]. We speculate that there is synergy between standing and positive lower body pressure which would cause local hypoxia increasing muscle perfusion and oxygen uptake [56, 57] as in pre-conditioning, thus potentiating the effects of the low intensity exercise improving mitochondrial function. Together with the described effects on the different classes of peptides, we posit that lower-body positive pressure increases parasympathetic tone in the cranio-sacral division of the autonomic nervous system, also reducing dysautonomia. Although not studied in these subjects ample evidence supports effects of LBPP on sympathetic tone, hemodynamics, oxygenation and metabotropic molecules [34, 38, 40, 41, 49, 50, 53, 56].

### Public health perspective

A recent review of obesity identifies that “decreasing time spent in occupational physical activities and displacement of leisure-time physical activities with sedentary activities” contributes to epidemic obesity. “Lack of effective and accessible life-style programs that can be administered locally or remotely at low cost to diverse populations” explain why “only a fraction of patients for whom treatments are indicated actually receive them” [3]. Among those that are treated, only a minority complete programs and fewer still sustain benefits [5, 6].

Our findings imply that it is possible to improve metabolic fitness in underserved populations with high prevalence of diabesity, barriers to exercising and cultural resistance to losing weight. The study coincides with reports of adverse effects of outdoor exercise in inner-city environments [58]. The indoor exercise in this study is provided by a safe and convenient weight-supporting lower-body pressure treadmill proven effective for ambulation in orthopedic and neurologic rehabilitation.

Effects of this magnitude on hyperinsulinemia, plasma triglycerides and ghrelin are exhibited after more time-consuming and intense volitional life-style changes or after metabolic operations recommended for type 2 diabetes [59]. The cost of an anti-gravity treadmill is well within the range of these modalities. This treadmill is safe and user-friendly and does not need supervising staff. Furthermore, it requires substantially less personal investment of time to use and is cost-effective since a single treadmill can be used by many subjects (e.g. in a family, therapeutically or preventively) over a long period, thus providing return on the initial investment regardless of payer.

### Limitations

This within-subject before-after study has no controls. The premise for using a body weight-supporting, lower-body positive pressure “anti-gravity” treadmill in people with chronic obesity is that walking at a normative pace (for able-bodied women of the same age) is painful and hard for obese subjects. They cannot and will not painlessly and effortlessly engage in walking on a conventional treadmill or, indeed, walking as an activity of daily living, unless paid or convinced of the utility of doing so in conflict with their own experiences (and extant published evidence). Recruiting such subjects is not ethically compatible with equipoise, where both investigator and subject are cognizant of the lack of efficacy of the “control” condition.

Limitations are having a *selected population* of volunteering, obese, *female* hospital workers of Caribbean-Black ancestry not allowing generalization to other populations with different BMI standards and metabolic metrics. However, regardless of geographic heritage our subjects are reproductive-age obese, relatively low socio-economic status (SES) women, representative of a large stratum of prevalently obese populations. Nevertheless, we must emphasize that our findings are limited to women, given well-documented sex differences in the proteomics. This relatively small exploratory study of a convenience sample of volunteers will require replication in larger more diverse populations and in men.

Our *variances* are substantial, reflecting “real-world” clinical research in our environment with significant confounders, yet achieve statistical significance with trends consistent with exercise studies in very different populations and designs (from trained Caucasian male athletes to exhaustive sub-maximal acute studies). We were unable to coordinate pre- and post-exercise blood sampling with our subjects’ *follicular phase*, which likely also has contributed to the variance. Time of day was variable for practical reasons. We did not detect any seasonality; the exercise was performed in a temperature-controlled gait laboratory, but we did not measure body temperature.

By design we omitted metrics of diet (macronutrient distribution, 3-day diaries), appetite (hunger ratings, taste preference), size and body composition (weight, BMI, lean body mass) and physical performance (O_2_ consumption, lactate production) to avoid imposing biases and leaving these metrics to future studies. There might have been changes in “dietary factors” although we doubt that such changes would occur spontaneously within 11 weeks and with only two 30-minute bouts/week walking 4 miles per hour [60].

We gave no dietary instructions or recommendations and when asked by subjects told them to “continue as usual”, although as mentioned some volitionally restricted their intake toward the end of the study and after completion.

This is not a conventional exercise study designed to improve physical fitness/performance; we did not collect such data. It is highly unlikely that 30 min of walking at a comfortable pace without exertion twice weekly for 10–12 weeks would measurably change VO_2_ max (a max performance test), just as it would not measurably change body composition, such as decreasing body fat or increasing lean body mass sufficiently to explain improved glucose disposal or decreased plasma triglycerides.

We have not included repeat post-study questionnaires given before to assess stress, sleep, mood, quality of life etc. owing to the short duration of this pilot study seriously affecting re-test reliability of instruments with significant variances requiring larger populations to detect significant differences. However, as mentioned earlier all subjects spontaneously expressed their enjoyment of this exercise, and have since volunteered for more studies.

The unique nature of this study, using a novel, thus uncommon, FDA approved publicly available device by unsolicited volunteering, self-selected hospital employees from a special inner-city population may be construed as reflecting “selection bias”. Therefore, we have taken great pains to emphasize the pilot nature of this exploratory hypothesis-generating study requiring replication in other more diverse populations and environments.

## Conclusions

We conclude that weight-supported lower-body positive pressure enables low intensity walking in subjects otherwise not inclined to exercise who hereby experience beneficial cardio- and neurometabolic effects without pain. This level of energy expenditure, in the sedentary range, is unlikely to trigger known counter-regulatory mechanisms induced by glycogen depletion common to moderate-high intensity exertion of longer duration recommended in public health guidelines for weight loss and prevention of diabetes and co-morbidities. Amelioration of the dysautonomia of allostatic load is an explanatory candidate mechanism, supported by numerous pre-clinical and clinical studies of effects of exercise and lower-body pressure stimulating the parasympathetic nervous system, modulating cardiac autonomic function [61], central neurogenesis and cognitive function [62, 63] associated with mood.

## Supporting information

S1 Table Characterization of 16 Caribbean-Black women. (DOCX)

S2 Table Pre- post- changes in selected gluco-regulatory, lipid and inflammatory molecules. (DOCX)

S1 Methods Daily energy expenditure. (DOCX)

S2 Methods Blood chemistry. (DOCX)

S1 Fig Lower-body positive pressure treadmill (AlterG). (DOCX)

S2 Fig A. Fasting s-triglyceride and B. p-ghrelin pre- and post- weight supported exercise.
(TIF)

S1 Dataset (ZIP)
