# Supplementary material for: A pilot study of metabolic fitness effects of weight-supported walking in women with obesity
Source: PLoS One. 2019 Feb 20;14(2):e0211529. doi: 10.1371/journal.pone.0211529 (PMC6382100; doi:10.1371/journal.pone.0211529)
Supplement: S1 Table — (DOCX) [file pone.0211529.s001.docx]

**S1 Table**  **Characterization of 16 Caribbean-Black women** (mean ± SD, except as noted).

Age (years) 39.7 ± 10.9

Weight (kg) 93.9 ± 13.6

Body mass index (kg•m^-2^) 34.9 ± 6.7

Waist circumference (cm) 108 ± 22

Blood pressure; SBP/DBP (mmHg) 113 / 74

p- Glucose (mmol•liter^-1^) 4.98 ± 0.88

p- Triglycerides (mmol•liter^-1^) 0.81 ± 0.30

p- Cholesterol (mmol•liter^-1^) 3.96 ± 0.78

p- HDL-Cholesterol (mmol•liter^-1^) 1.2 ± 0.28

p- Insulin (pmol•liter^-1^) 92.3 ± 59.5
