# Supplementary material for: A pilot study of metabolic fitness effects of weight-supported walking in women with obesity
Source: PLoS One. 2019 Feb 20;14(2):e0211529. doi: 10.1371/journal.pone.0211529 (PMC6382100; doi:10.1371/journal.pone.0211529)
Supplement: S2 Table — (DOCX) [file pone.0211529.s002.docx]

**S2 Table Pre- post- changes in selected gluco-regulatory, lipid and inflammatory molecules** (mean ± SD)**.**

**Pre- Post- Change p**

C-peptide nmol•liter^-1^ 0.39 ± 0.16 0.37 ± 0.133 -0.019 ± 0.094 0.44

HDL-cholesterol mmol•liter^-1^ 1.20 ± 0.28 1.22 ± 0.22 0.02 ± 0.195 0.82

Glucagon pmol•liter^-1^ 5.66 ± 2.38 5.22 ± 3.07 - 0.44 ± 3.9 0.65

GIP pmol•liter^-1^ 9.39 ± 4.92 9.15 ± 5.36 - 0.24 ± 5.65 0.86

GLP-1 pmol•liter^-1^ 18.84 ± 41.6 18.77 ± 7.51 -0.065 ± 7.69 0.98

Free Fatty Acids mmol•liter^-1^ 0.53 ± 0.23 0.54 ± 0.24 0.011 ± 0.35 0.90

C-Reactive Protein mg•liter^-1^ 6.8 ± 5.0 6.3 ± 4.7 -0.48 ± 3.71 0.54

TNF-α pg•ml^-1^ 3.0 ± 1.5 3.1 ± 1.4 0.1 ± 0.4 0.23
