# Supplementary material for: A pilot study of metabolic fitness effects of weight-supported walking in women with obesity
Source: PLoS One. 2019 Feb 20;14(2):e0211529. doi: 10.1371/journal.pone.0211529 (PMC6382100; doi:10.1371/journal.pone.0211529)
Supplement: S1 Methods — (DOCX) [file pone.0211529.s003.docx]

**S1 Methods Daily energy expenditure**  Equation for correcting MET values from the Compendium of Physical Activities using estimated RMR**
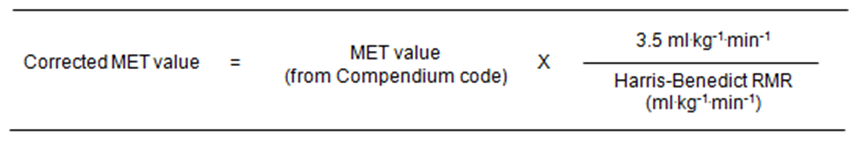
**

ACSM guidelines recommend 500-1000 MET minutes per week corresponding to 150 minutes of moderate activity per day, consistent with a step count greater than 5400-7900 per day. Using 3-day FitBit® accelerometer records we calculated that participants exhibited a “low” to “moderate” level of activity based on ACSM guidelines of number of miles or steps taken per day. Our participants walked on average 3 miles /day. Calculated at a typical cadence of walking 100 steps/min (normal cadence up to117 steps /min), 1 mile at this moderate estimate of intensity equals approximately 2000 steps. Three miles corresponds to 6000 steps, 40% lower than the mean recommended step count for weight management of 10,000/day.
