# Supplementary material for: A pilot study of metabolic fitness effects of weight-supported walking in women with obesity
Source: PLoS One. 2019 Feb 20;14(2):e0211529. doi: 10.1371/journal.pone.0211529 (PMC6382100; doi:10.1371/journal.pone.0211529)
Supplement: S2 Methods — (DOCX) [file pone.0211529.s004.docx]

**S2 Methods Blood chemistry**

Plasma glucose, C-reactive protein (CRP), fasting lipid panel and free fatty acids (FFA) were analyzed at SUNY Downstate Medical Center hospital laboratory. Singleplex or Multiplex cytokine/chemokine plasma analyses and ELISA of GIP, glucagon, IL-6, leptin, TNF-α, GLP-1, C-peptide, insulin, total adiponectin, total ghrelin, β-endorphin, orexin-A (ORA), and oxytocin (OXT) were performed by the Human Immune Monitoring Core Facility (HIMC) of Mount Sinai School of Medicine, New York using Luminex xMAP® [EMD Millipore Corporation, Billerica, Mass, USA] . Plates were read using a molecular devices plate reader [Bioplex 200 with Bioplex Manager (Biorad, Hercules, CA)] and data analysis was performed using Softmax Pr0o 5.0.

Coefficients of variance (CV %) and sensitivity (sens.) or minimum detection concentration in pg/ml:

[1. Metabolic Hormone Magnetic Bead Panels - Metabolism Multiplex Assay](https://www.emdmillipore.com/US/en/product/MILLIPLEX-MAP-Human-Metabolic-Hormone-Magnetic-Bead-Panel---Metabolism-Multiplex-Assay,MM_NF-HMHEMAG-34K): *GIP, Glucagon, IL-6, Leptin, TNF-Alpha***:** CV 0.35%, 0.24%, 0.48%, 0.43%, 0.26%, sens**.** 0.40 pg/ml, 6.46pg/ml, 4.94 pg/ml, 12.77 pg/ml, 0.20 pg/ml (# HMHEMAG-34K-05**)** and *GLP-1, C-peptide, insulin***:** CV 1.98%, 0.10%, 0.43% sens. 2.12 pg/ml, 6.36 pg/ml, 61.14 pg/ml (#HMHEMAG-34K-03y).

2. Neuropeptide Magnetic Bead Panel - Neuroscience Multiplex Assay: *Beta endorphin, orexin-A, oxytocin*: CV 0.13%, 0.36%, 0.078%, sens. 45.82 pg/ml, 81.15 pg/ml, 7.32 pg/ml (# HNPMAG-35K-03).

3. Adipokine Magnetic Bead Panel - Singleplex Assay: *total adiponectin*: CV 0.31% sens. 8.8 pg/ml (#HADK1MAG-61K-01).

4. *Total ghrelin* was measured using ELISA: CV 0 %, sens. 30 pg/ml (#EZGRT-89K).
