# Supplementary figures and images for: A pilot study of metabolic fitness effects of weight-supported walking in women with obesity
Source: PLoS One. 2019 Feb 20;14(2):e0211529. doi: 10.1371/journal.pone.0211529 (PMC6382100; doi:10.1371/journal.pone.0211529)

**S1 Figure**


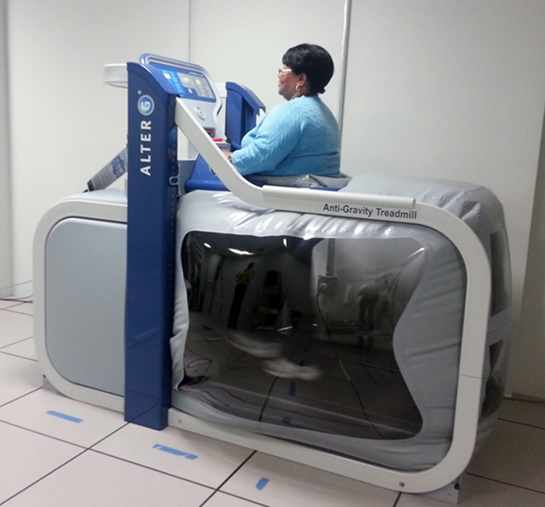

Supplement: S1 Fig — (DOCX) [file pone.0211529.s005.docx]
